# The small heat shock protein (sHSP) genes in the silkworm, *Bombyx mori*, and comparative analysis with other insect sHSP genes

**DOI:** 10.1186/1471-2148-9-215

**Published:** 2009-08-28

**Authors:** Zi-Wen Li, Xue Li, Quan-You Yu, Zhong-Huai Xiang, Hirohisa Kishino, Ze Zhang

**Affiliations:** 1 The Key Sericultural Laboratory of Agricultural Ministry, Southwest University, Chongqing 400715, PR China; 2 The Institute of Agricultural and Life Sciences, Chongqing University, Chongqing 400044, PR China; 3 The Laboratory of Biometrics and Bioinformatics, Graduate School of Agriculture and Life Science, The University of Tokyo, 1-1-1, Yayoi, Bunkyo, Tokyo, 113-8657, Japan

## Abstract

**Background:**

Small heat shock proteins (sHSPs) are products of heat shock response and of other stress responses, and ubiquitous in all three domains of life, archaea, bacteria, and eukarya. They mainly function as molecular chaperones to protect proteins from being denatured in extreme conditions. Study on insect sHSPs could provide some insights into evolution of insects that have adapted to diverse niches in the world.

**Results:**

Taking advantage of the newly assembled genome sequence, we performed a genome-wide analysis of the candidate sHSP genes in the silkworm, *Bombyx mori*. Based on known silkworm sHSP sequences, we identified 16 silkworm sHSP genes. Most of them are distributed on two silkworm chromosomes 5 and 27, respectively. 15 of 16 silkworm sHSPs have expression evidence. The comparative analysis of insect sHSPs from *B. mori*, *Drosophila melanogaster*, *Apis mellifera*, *Tribolium castaneum*, and *Anopheles gambiae *revealed that there is only one orthologous cluster whereas remaining clusters are species-specific on the phylogenetic tree. This suggested that most of sHSPs might have diverged in function across insects investigated. In addition, the data presented in this study also revealed that sHSPs in the insect orthologous cluster are highly conserved in both sequence and expression pattern. In sum, insect sHSPs show a completely different evolutionary pattern from that found in vertebrate sHSPs.

**Conclusion:**

*B. mori *has the largest number of insect sHSP genes characterized to date, including 16 genes. The inference that most species-specific sHSPs might have diverged in function across insects investigated will help us understand the adaptability of these insects to diverse environments.

## Background

It was in *Drosophila *that researchers observed the phenomenon of heat shock response (HSR) at the first time [1]. Then these proteins highly expressed in thermal stress were defined as heat shock proteins (HSPs). Previous studies suggest that the small heat shock proteins (sHSPs) are abundant and ubiquitous in almost all organisms, from bacteria to algae with single cell to the higher organisms including human [2-5]. HSPs can be divided into five families, including HSP100, HSP90, HSP70, HSP60, and sHSP. This classification is mainly based on the molecular weight as well as the homologous relationship of HSPs. All of these families are conserved, except for sHSP family that is more diverse than other four. However, there are some common characteristics in the function and structure of sHSP. The molecular weights of sHSPs range from 12 kDa to 42 kDa, usually bellow 30 kDa [2,4]. The sHSPs have an α-crystalling domain comprising about 100 amino acid residues, which is the conserved structure of all sHSP sequences [6-8]. There is a conserved β-sheet sandwich in sHSP secondary structure, and it is these β-sheet structures that help several subunits of sHSP to form a large oligomer [2,9,10]. This stable multimeric structure formed by sHSPs has the function of molecular chaperone, which binds to the proteins and prevents them from thermal denaturation [10-12]. The low molecular weight, the conserved secondary structure, and protein domain are principal features used to identify a protein belonging to sHSP family.

In addition to functioning as molecular chaperones to protect proteins from being denatured in high temperature stress [10,13], sHSPs can also develop the protection function in the conditions of other stresses, such as cold, drought, oxidation, hypertonic stress, UV, and heavy metals [4,14], even high population density of organisms [15]. The HSPs including sHSPs are playing the part of chaperone function not only in stress conditions, but also in normal development [13]. Although the sHSP family has relatively conserved function of molecular chaperone and the C-terminal of these proteins harbors the conserved α-crystalling domain, the N-terminal sequences of these proteins are variable. This indicates that the conserved C-terminal sequence is a significant part for sustaining the chaperone and other functions of sHSP in cells or organisms whereas the diverse N-terminal sequences may be associated with the diverse expressions, functions, and evolutionary patterns among sHSPs.

There have been numerous studies on HSPs in bacteria, algae, plant, amphibians, birds, and mammalian, especially in the model organisms of *Arabidopsis thaliana*, *Saccharomyces cerevisiae*, *Caenorhabditis elegans*, *Drosophila melanogaster*, *zebra fish*, *Mus musculus*[3-5]. Although the heat shock response was fist discovered in insect [1], the study of HSP in insects, especially for sHSP, is not as extensive and penetrating as in other organisms. sHSPs primarily have chaperone activity and reflect the response machine of organisms to some extreme stresses existing in environment. Insects are one of the most successful organisms in the world and have a strong ability to adapt to various habitats. So the study of insect sHSP is necessary.

In this study, we first identified the sHSP genes of *Bombyx mori *based on the new assembly of the silkworm genome sequence [16]. Then we performed comparative analyses with the sHSPs from other four insects whose complete genome sequences are available. It was found that there is an orthologous cluster including five sHSP genes that come from each of five insects. This group of genes is so conserved in sequence among insects, which is in sharp contrast to the other sHSP genes that are species-specific and show the evolutionary pattern of lineage-specific expansion. In addition, the expression patterns of the silkworm sHSPs were investigated by available microarray data. Our data provide some new insights into evolution and functions of the insect sHSPs.

## Results

### sHSP genes in silkworm and other insects

Previous studies have reported 7 silkworm sHSP genes [17-19]. Using these known sHSP sequences as queries, in total we identified 16 sHSP genes in silkworm through homology search (Table 1). The molecular weight of each silkworm sHSP gene was predicted by the online tool of ExPASy website. Finally, we named these genes using their predicted molecular weights, respectively.

**Table 1:** The sHSP genes in *B. mori*

Gene	Gene ID	Chromosome location	Scaffold and Interval	Intron number	Length (bp)	Predicted molecular weight (Da)	Reference
Bm23.8	BGIBMGA004515-TA	27	nscaf2797 (-):1074340..1074975	None	636	23757.91	Sakano et al.2006
Bm20.8	BGIBMGA004605-TA	27	nscaf2800 (+):2702345..2702905	None	561	20804.46	Sakano et al.2006
Bm20.4	BGIBMGA004541-TA	27	nscaf2800 (-):2697134..2697679	None	546	20427.07	Sakano et al.2006
Bm19.9	BGIBMGA004540-TA	27	nscaf2800 (-):2713425..2713958	None	534	19890.48	Li et al.2005
Bm19.1	BGIBMGA004606-TA	27	nscaf2800 (+):2709883..2710389	None	507	19057.44	
Bm15.7	Bmb030089	27	nscaf2836 (+):272788..273204	None	417	15686.01	
Bm42.3	BGIBMGA004101-TA	19	nscaf2767 (+):1827016..1828122	None	1107	42317.16	
Bm22.6	BGIBMGA004103-TA	19	nscaf2767 (+):1871338..1874916	one	597	22561.50	
Bm21.4	BGIBMGA000944-TA	13	nscaf1898 (-):9135882..9142328	two	564	21403.99	Sakano et al.2006
Bm27.4	BGIBMGA005823-TA	5	nscaf2838 (+):1796002..1796742	None	741	27417.37	
Bm26.6	BGIBMGA005755-TA	5	nscaf2838 (-):1724199..1724924	None	726	26586.88	
Bm24.2	BGIBMGA005780-TA	5	nscaf2838 (-):44799..45431	None	633	24221.60	Liu et al.2008
Bm20.2	BGIBMGA005784-TA	5	nscaf2838 (+):41711..42247	None	537	20200.98	
Bm19.5	BGIBMGA013545-TA	5	nscaf3076 (+):59355..59858	None	504	19535.26	
Bm21.6	BGIBMGA004630-TA	Unknown	nscaf2803 (+):14722..15288	None	567	21620.41	
Bm20.1	Bmb016799	Unknown	scaffold2784 (-):298..834	None	537	20138.60	Sakano et al.2006

**Figure 1:**
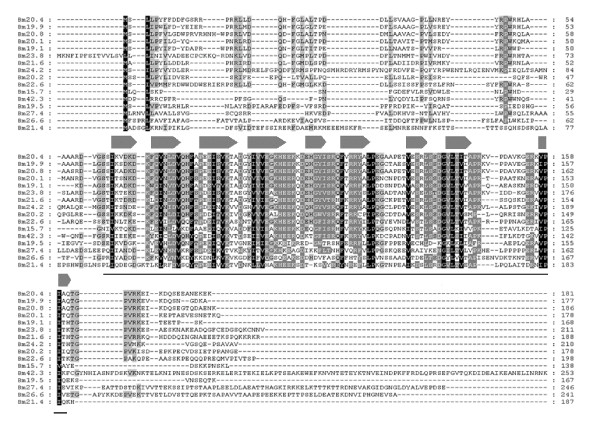
**The alignment of silkworm sHSP sequences and their secondary structure and domain**. Sequences under black arrows are regions of β-sheet sandwich structure, which were numbers with β2-β10 from N-terminal to C-terminal, respectively. Sequences above black line are regions of α-crystalling domain.

The sHSP genes have conserved protein secondary structure and functional domain [13]. The molecular weight of sHSP is usually between 12 kDa to 42 kDa [2]. There is an α-crystalling domain comprising of about 100 amino acid residues in secondary structure of silkworm sHSPs [6-8]. Silkworm has 16 sHSP genes, which is the largest number in five insects investigated. α-crystalling domain harbors 9 β-sheet sandwich structures (Figure 1). These β-sheets are numbered with β2 to β10 (Figure 1) which correspond to the secondary structure of sHSP16.9 in wheat, *Triticum aestivum *[9] and sHSP25 in *Ca. elegans *[5]. These features in sequence structure can be used to identify the candidate genes. It will be seen from Figure 1 that the candidates identified in this study are indeed silkworm sHSP genes. Compared with the conserved structure of C-terminal, the N-terminal regions of silkworm sHSP genes are relatively divergent in sequence length and order. However, there are still some conserved amino acid residues in N-terminal.

*B. mori *has 28 chromosome pairs. It can be known from Table 1 that the genes encoding sHSPs were distributed on at least four chromosomes 27, 5, 19, and 13. Because the scaffolds nscaf2803 and scaffold2784 could not be located on the chromosomes in current version of the silkworm genome sequence, the locations of Bm21.6 (in nscaf2803) and Bm20.1 (in scaffold2784) were not determined. It is clear that the distribution of the silkworm sHSP genes in the genome is not homogeneous. There are six sHSP genes located on the silkworm chromosome 27, five genes on chromosome 5, two genes on chromosome 19, and only one gene on chromosome 13. In addition, we found that genes distributed on the chromosomes 27 and 5 have no intron. One gene, Bm22.6 distributed on chromosome 19, contains one intron (the intron length is 2982 bp), and another one, Bm21.4 distributed on chromosome 13, contains two introns (the lengths are 5263 bp and 620 bp, respectively). Compared with the full-length (400~750 bp) of most of the silkworm sHSP genes, these two genes containing introns are relatively too long, especially for Bm21.4. In the previous report [18], the complete gene sequence of Bm21.4 could not be amplified through PCR method using silkworm genomic DNA as template. Probably, this is due to existence of a relatively big intron. Bm22.6, Bm21.4, and Bm42.3 are different from the other silkworm sHSP genes in chromosome location and the existence of intron. It can be seen from Table 1 that Bm20.8, Bm20.4, Bm19.1, and Bm19.9 are tandem arranged on the middle region of chromosome 27 and there is no any other gene interrupting this genomic region. Bm 23.8 is located on the near end of chromosome 27. However, only two of five sHSP genes on chromosome 5 are tightly tandem arranged (Table 1 and Additional file 1).

The sHSP genes of other four insects, *D. melanogaster*, *Ap. mellifera*, *T. castaneum*, and *An. gambiae*, are listed in Table 2. We renamed these genes with their molecular weights. It was found that *D. melanogaster *has 11 sHSP genes, *Ap. mellifera *and *T. castaneum *have 10 genes, respectively, and *An. gambiae *has 7 genes. The distribution patterns of the sHSP genes in the genomes of four insects are similar to that in the silkworm genome. That is, most of sHSP genes in each organism are located on one chromosome, for example, chromosome 3 of *D. melanogaster *(8 sHSP genes), chromosome 2 of *Ap. mellifera *(8 genes), chromosome 8 of *T. castaneum *(7 genes), and chromosome 2 of *An. gambiae *(6 genes). The remaining two or three sHSP genes are distributed on other chromosomes. Furthermore, most sHSP genes located on one chromosome are usually tandem arranged, and the remaining one or two sHSP genes are separately distributed on the same chromosome with some distances from the tandem set (Table 2 and Additional file 1). Although the number of tandem arranged genes varies among the insects, the chromosomes harboring most sHSP genes in each species exhibit a similar gene distribution pattern.

**Table 2:** The sHSP genes in other four insects

Gene	Accession number	Chromosome location	Intron number	Predicted molecular weight (Da)	Length (bp)
*Drosophila melanogaster*					
				
Dmel20.8	NM_134482	X(+):19499191..19505226	Three	20841.58	552
Dmel20.6	NM_079275	3L(+):9374984..9375867	None	20629.33	561
Dmel23.0	NM_079273	3L(-):9369518..9370527	None	22994.14	627
Dmel23.6	NM_079276	3L(+):9377165..9378384	None	23616.62	642
Dmel22.2	NM_079270	3L(-):9364818..9365417	None	22179.99	600
Dmel18.0	NM_139898	3L(+):7747509..7748274	None	17966.28	465
Dmel19.8	NM_001031943	3L(+):9366031..9368070	None	19763.26	525
Dmel23.8	NM_140047	3L(+):9368529..9369369	None	23777.81	603
Dmel46.9	NM_079274	3L(-):9370902..9372634	None	46930.96	1338
Dmel21.3	NM_079103	2R(-):19572174..19573063	Two	21308.98	564
Dmel24.5	NM_135499	2L(-):10091582..10092235	None	24538.17	654
*Apis mellifera*					
				
Amel25.6	XM_392405	6(+):13993929..14019055	Three	25565.47	684
Amel23.0	XM_001120194	2(+):3399670..3400530	None	23001.99	591
Amel22.6	XM_001119884	2(+):3385350..3386366	None	22560.49	585
Amel22.0	XM_394333	2(+):11463525..11474444	Three	21975.63	576
Amel22.5	XM_001120070	2(+):3393512..3394262	None	22462.40	582
Amel21.3	XM_393575	2(-):3387271..3388175	One	21282.19	546
Amel24.2a	XM_001119830	2(-):3382631..3384101	None	24239.70	633
Amel27.7	XM_001120137	2(+):3396138..3397264	None	27697.70	714
Amel24.2b	XM_001120006	2(+):3391125..3392082	None	24211.50	632
Amel20.4	XM_395659	1(+):16200748..16202481	One	20446.12	525
*Tribolium castaneum*					
				
Tcas20.8	XM_969297	8(+):17781223..17781771	None	20800.60	549
Tcas20.7a	XM_968349	8(-):14292594..14293142	None	20695.49	549
Tcas20.7b	XM_961687	8(+):14295428..14296012	None	20734.30	540
Tcas18.3	XM_969274	8(-):17779083..17779699	None	18342.49	474
Tcas19.7	XM_968251	8(-):14288920..14289435	None	19721.01	516
Tcas16.0	XM_968285	8(-):14289936..14290352	None	16017.01	417
Tcas13.2	XM_968317	8(+):14290622..14290975	None	13228.48	354
Tcas21.8a	XM_963667	7(+):17190509..17191251	One	21828.46	579
Tcas22.2	XM_970284	3(+):8758863..8759450	None	22238.23	588
Tcas21.8b	XM_968592	2(+):14516743..14518763	Three	21785.43	573
*Anopheles gambiae*					
				
Agam20.9	XM_560153	X(-):18244471..18251117	Two	20945.63	564
Agam21.6	XM_308606	2L(-):43612360..43613412	Two	21622.20	579
Agam23.7	XM_308609	2L(-):43600970..43601852	None	23717.49	624
Agam23.5a	XM_315549	2L(+):16952069..16952914	None	23492.29	621
Agam23.4	XM_315550	2L(-):16955265..16956127	None	23355.15	618
Agam23.5b	XM_308607	2L(+):43610629..43611658	Two	23507.23	621
Agam21.7	XM_308608	2L(-):43603779..43607408	Three	21691.67	555

Silkworm has the largest number of sHSP genes (16 genes) whereas *An. gambiae *has the least number of sHSP genes (7 genes) among the insects investigated. With the information of chromosome location and intron number, we can roughly divide the sHSP genes into two types for each of the species. One is that the genes clustered on the same chromosome (two chromosomes for silkworm) tend to have no intron and the other is that the genes (1 to 3 genes) dispersed on chromosomes tend to have intron.

We also performed homology search in the genome of *S. cerevisia *and found two genes that have high identity with silkworm sHSP genes. One is Scer23.9, located on the chromosome 2. This gene has been reported previously and its NCBI accession number is NP_009628.1. We used it as the outgroup in the phylogenetic analyses.

### Phylogenetic analysis of insect sHSP genes

**Figure 2:**
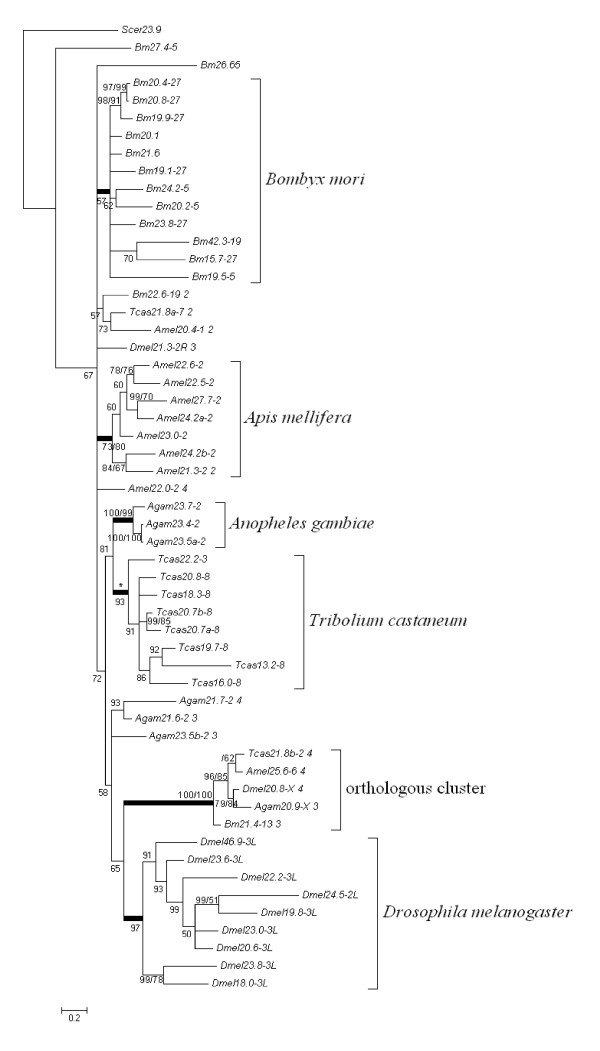
**The Bayesian tree of conserved sHSP amino acid sequences of *B. mori, D. melanogaster, An. gambiae, Ap. Mellifera, and T. castaneum***. Numbers beside the nodes indicate the supporting values measured by the posterior probabilities. The names of amino acid sequences are composed of species names and predict molecular weights. The numbers behind dashes are linkage groups on which the genes are located. If there is another number following the linkage group, it is the number of exons for that gene. We also reconstructed an NJ tree with JTT amino acid matrix and pairwise deletion in MEGA, using the same alignment sequences. The bootstrap values (1000 bootstrap replicates) are listed following the posterior probabilities. The wide branches indicate that the gene members behind them are the same between the Bayesian tree and the NJ tree. However, Tcas13.2 marked with asterisk is not behind the corresponding branch in the NJ tree.

Programs MrBayes v.3.1.2 and MEGA 4.0 were used to reconstruct Bayesian and NJ phylogenetic trees for the insect sHSP genes. The topologies of phylogenetic trees obtained by different approaches are similar. So, only the Bayesian tree is shown in Figure 2 (additional file 2).

Figure 2 shows that there are two types of clusters on the phylogenetic tree. One type is an orthologous cluster with a very high supporting value measured by the posterior probability, which includes five members that come from each of five insects, respectively. These genes all have at least two introns and dispersed in genomes without any linkage relationships to other sHSP genes. The sHSP genes of *D. melanogaster *(Dmel20.8) and *An. gambiae *(Agam20.9) are located on the chromosome X, which is the sex chromosome. The branching pattern of the orthologous cluster shows that Dmel20.8 and Agam20.9 are closer in five orthologous genes and Amel25.6 and Tcas21.8b are closer. Surprisingly, Bm21.4 did not cluster closely to Diptera but was placed on the basal of the cluster, which is not consistent with the result of a recent study on insect molecular systematics [20]. This may imply that Bm21.4 might have experienced a specific evolutionary process.

The other type on the phylogenetic tree is species-specific clusters as a result of lineage-specific expansion. Silkworm-specific cluster includes 12 sHSP genes but has a relative low support value. Most of these genes are located on two chromosomes 5 and 27, however, Bm27.4 and Bm26.6 located on chromosome 5 are not included in this cluster. Bm42.3 is also contained in this cluster.

The posterior probabilities of the species-specific clusters in other four insects are higher (Figure 2). In *Ap. mellifera*, the species-specific cluster contains 7 sHSP genes and all these genes are distributed on chromosome 2. Additionally, 6 of these genes have no intron except Amel21.3 contains one intron. It is interesting that Amel22.0 located on chromosome 2 and with three introns is not the member of this cluster. In *D. melanogaster*, sHSP genes distributed on the left arm of chromosome 3 are all included in the species-specific cluster. The gene of Dmel24.5 located on chromosome 2 is also in this cluster. All these genes have no intron. Most of members of the species-specific cluster of *T. castaneum *sHSP are located on chromosome 8 and only Tcas22.2 is on chromosome 3. They are all single exon genes. Three sHSP genes on the same chromosome of *An. gambiae *formed a cluster and they all have no intron. In addition, there are other sHSP specific genes in *An. gambiae*, including Agam21.7, Agam21.6, and Agam23.5b. These three genes have two or three introns and are also located on chromosome 2. All tandem arranged genes of each species except *An. gambiae *are included in the species-specific cluster (Table 1, Table 2 and Figure 2).

Most strikingly, most members in each of species-specific clusters are usually distributed on the same chromosome in a tandem manner. This gene expansion may be the result of gene duplication events created by unequal crossing over.

### The orthologous sHSP genes in insects

**Figure 3:**
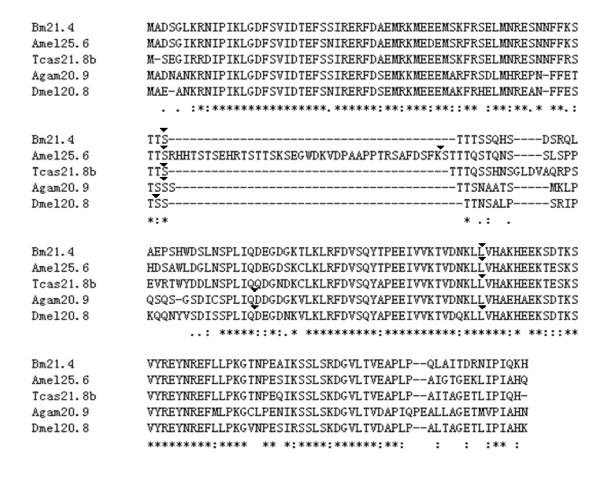
**The alignment of the amino acid sequences of orthologous sHSP genes from five insects**. The triangles show positions of corresponding introns. Asterisk represents identical residues.

The phylogenetic analysis of sHSP genes in five insects reveals an orthologous cluster (Figure 2). This cluster contains five sHSP genes corresponding to five insects, respectively (Figure 3). These sHSPs have similar sequence lengths of 183 to 190 amino acid residues except that Amel25.6 has an extra and distinct exon containing 38 amino acid residues. The amino acid sequences of these orthologous sHSPs are highly conserved not only in the C-terminal region of α-crystalling domain with chaperone function but also in the N-terminal sequences that are more variable in other sHSP genes (Figure 1 and 3). The five conserved sHSP genes all have introns: Bm21.4 and Agam20.9 have two introns and Dmel20.8, Tcas21.8b, and Amel25.6 have three. Generally speaking, there are four intron positions in the alignment of the orthologous sHSP gene sequences. Intron 1 is conserved in these five genes and has phase 2. Intron 2 only exists in honeybee orthologous sHSP and also has phase 2. Intron 3 is special for *D. melanogaster *and *An. gambiae*, both are Diptera. Intron 4 is also conserved in orthologous sHSP genes of these insects except *An. gambiae *which has no intron in this position. Given that the orthologous sHSP genes have the conserved features in sequence, the intron position as well as the special and distinct phylogenetic relationships with other sHSP genes, we suppose that these sHSP genes are conserved in function across insects. They may play important roles in basic biological processes.

We performed blast searching Genbank with these orthologous sHSP sequences to look for orthologous sHSPs in other insects or distant species. We did find that many other insects have this orthologous gene, such as *Acyrthosiphon pisum *(Accession no. XP_001949402), *Aedes aegypti *(XP_001657982), *Culex quinquefasciatus *(XM_001870731), *Heliconius erato *(ABS57447), *Locusta migratoria *(ABC84493), *and Nasonia vitripennis *(XP_001607669). Furthermore, an arthropods of *Ixodes scapularis *(EEC06453) belonging to Arachnoidea and four nematoda organisms of *Caenorhabditis briggsae *(XP_001676833), *C. elegans *(NP_001024374), *Brugia malayi *(U48407) and *Dirofilaria immitis *(AAB08736) also contain the orthologous sHSP genes which are slightly divergent from the insect orthologs. Compared with the five orthologous sHSP genes in Figure 2, the homologous genes mentioned above have not only high identity but also a conserved N-terminal, which is the principal structure characteristic of the genes in the orthologous cluster (Additional file 3). So, this kind of orthologous genes exists not only in insects but also in other invertebrates.

To confirm whether the orthologous group genes exist in vertebrate, we reconstructed a larger phylogenetic tree including sHSP genes from above five insects, nematode, amphioxus, mouse and human (Additional file 4). The sequences of sHSP genes in nematode, mouse and human came from previous studies [5,21,22]. 14 amphioxus sHSP genes were obtained by searching the genome of *Branchiostoma floridae *[23] using blast method. The phylogenetic analysis results indicated that, as expectation, a nematode sHSP gene Ce25 was included in the highly supported orthologous cluster but the human and mouse sHSP genes were not in it. However, the HsapHspB11 from Human and MmusHSPB11 from mouse as well as two sHSP genes from amphioxus formed a cluster although the support value is low. Furthermore, this cluster seems to be close to the orthologous group (Additional file 4). Therefore, it appears that a kind of highly conserved sHSP genes may exist even in vertebrates. In addition, we observed that there are many orthologous sHSP clusters between human and mouse, which is consistent with previous studies [21,22].

### The species specific sHSP genes in insects, especially in the silkworm

Our study revealed that most insect sHSP genes are species-specific (Figure 2). The silkworm contains the most number of the species-specific sHSPs. They all have a conserved α-crystalling domain and a diversified N-terminal region. Whether there are divergent expression patterns in these paralogous genes is worthy to be detected for this domesticated insect, *B. mori*.

We searched for EST evidences of sHSP genes through SilkDB [24]. 14 silkworm sHSP genes have complete EST sequences except Bm21.6 and Bm42.3. But Bm21.6 had been cloned in previous studies, as well as the genes of Bm19.9 [17,18], Bm23.8, Bm20.8, Bm20.4, Bm21.4, and Bm20.1 [18], Bm24.2 [19], Bm19.1, Bm15.7, Bm 22.6, Bm27.4, Bm20.2, and Bm19.5 (not published). So, 15 silkworm sHSP genes are certainly transcribed. In fact, there is no EST sequence associated with Bm42.3. Several possible reasons may explain the absence of EST evidence for Bm42.3. One is that this candidate silkworm sHSP gene is not expressed in usual conditions or its expression is too weak to be detected. Another is that it might have lost its regulatory elements. All of these should be confirmed in future.

**Figure 4:**
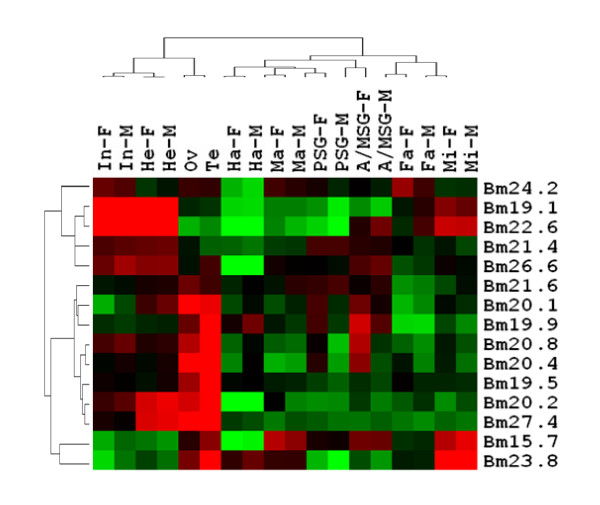
**The expression profile of silkworm sHSP genes in different tissues by microarray data**. Red indicates upregulated genes; green down-regulated genes; black no expression change. The tissues: In (integument), He (head), Ov (ovary), Te (testis), Ha (hemocyte), Ma (malpighii tuba), PSG (Posterior Silk Gland), A/MSG (anterior and middle silk gland), Fa (fat body), and Mi (midgut). "F" means female, and "M" means male.

We also investigated the expression patterns of sHSP genes in silkworm through the microarray data in BmMDB [25]. The database contains the expression information for more than twenty thousands genes in 9 silkworm tissues in day 3 of the fifth instar. However, all of the expression data were obtained in a normal condition. One candidate sHSP gene in silkworm, Bm42.3, is not available in the database. So there are 15 silkworm sHSP genes that have expression information of different tissues (Figure 4). It can be seen from Figure 4 that the male and female clustered together in each tissue, suggesting that the sHSP expression patterns of male and female were similar in most of the tissues. However, there are some female upregulated sHSP genes, implying the sexually distinct expression pattern in silkworm [25], for example, hsp20.8 and hsp19.9 in A/MSG, and hsp20.8, hsp20.1, hsp19.9 in the PSG (Figure 4). In addition, Bm19.1 and Bm22.6 had relatively high expression levels in integument, head and midgut. Bm20.1, Bm20.4 and Bm27.4 were highly expressed in gonad, either in testis or in ovary. These sHSP genes expressed in special tissues without stress may play an important role in keeping the normal functions of the cell and tissues.

## Discussion

In this study, we first identified 16 silkworm sHSP genes based on the new assembly of the *B. mori *genome sequence [16], which is the largest number of the sHSPs of five insects in different orders that whole genome sequences are available. Previous study based on six silkworm sHSP genes suggested that the sHSP genes in the silkworm may be classified into two groups [18]. Similarly, the comparative analysis of sHSPs in five insects revealed that there are two types of clusters of insect sHSPs: one is orthologous and the other is species-specific. Interestingly, there is one orthologous cluster that includes one sHSP from each insect whereas most insect sHSPs are species-specific (Figure 2). These results suggested that most insect sHSPs might have experienced lineage-specific expansion and diverged in function at least amongst insect orders. Nevertheless, a few insect sHSPs such as those in orthologous cluster are highly conserved in whole sequence as well as in gene structure, suggesting that they may also have highly conserved function. The phylogenetic relation of the orthologous cluster may be interpreted as the speciation history.

Most members of species-specific cluster are tandem arranged genes in each insect and there is no orthologous relationship between tandem genes of different insects. This case may occur if a primitive tandem set with several genes existed in the ancient of the five insects, subsequently, frequent and general gene conversion events homogenize the tandem sHSP genes in each insect after the speciation, finally the originally orthologous sHSP genes became species-specific. So we estimated the gene conversion events of sHSP genes in each insect with GeneConv program [26]. Surprisingly, although the *P *values are below 0.01, the predicted gene conversion nucleotide residues are no longer than 40 bp in our tests (most are below 25 bp) except a gene pair in the silkworm of Bm20.8 and Bm 20.4 (*P *value, 0.001; length, 128 bp). These results imply that gene conversion is not the major mechanism explaining the topology in Figure 2. Therefore, our observations do not support the assumption above. Members of species-specific cluster or tandem arranged genes might be the result of independent gene expansion occurred during evolution of each insect.

The gene expansion process may associate with gene duplication and loss events, which are known as a birth-and-death model. This model was successfully used to interpret some evolutionary process of most gene families including HSP70 and cytochrome P450 [27,28]. If this is the case, Figure 2 suggests that some gene duplication events of sHSPs occurred before the radiation of insects, and that one or two copies experienced lineage-specific expansions through successive gene duplications after the radiation of insects.

The tandem arranged pattern of sHSP genes is not exclusive in insects. For example, sHSP16 genes in *C. elegans *and *C. briggsae *also show similar pattern [5]. The tandem repeats of sHSP genes may facilitate organisms to rapidly respond to changing environmental conditions because of regulation reason. All the five insects have independently developed this strategy for sHSP genes, which is similar to the convergent duplication of the proneural genes in insects [29]. However, the 10 sHSP genes in human are separately distributed on eight chromosomes [21]. The different arranged forms of sHSP genes may reflect different evolutionary processes that organisms experienced. Tandem sHSP genes may be a better way for insects to regulate gene expressions in diverse environments.

It is known that the sHSPs are members of HSP family with function of molecular chaperone. They are ubiquitous in all organisms and extremely conserved in the sequence structure of α-crystalling domain resided in C-terminal. The conserved C-terminal region might be the result of strongly functional constraint of sHSPs as a molecular chaperone. sHSPs bind to other cellular proteins in thermal and other extremely devastating stresses, and protect them from denaturation. They also have functions in protein folding and transportation, the embryo development and immunization mechanism of organisms. The conservative degrees of sHSP genes are heterogeneous in different protein regions. In general, the N-terminal coding sequences are more variable than C-terminal with the α-crystalling domain. However, the sHSPs in the orthologous cluster showed high conservation along whole sequence. The orthologous sHSP gene (Bm21.4) in silkworm was cloned and found to be not induced by thermic stress (45°C), and it was expressed constitutively in normal conditions (room temperature) in fat body and other tissues of silkworm [18]. The microarray data also revealed the similar expression patterns (the probe number is sw01265). So Bm21.4 is special in sequence and expression compared with other silkworm sHSP genes [18]. These observations suggest that insect orthologous sHSPs may not be associated with response of insects to environmental stresses. In contrast, they may be involved in basically metabolic processes in insects.

One previous study based on the limited number of sHSP sequences suggested that the phylogenetic relationship of sHSP genes in insects is quite different from that of other species or taxonomical groups [6]. Mammals have at least ten sHSP subfamilies and they also have orthologous genes in zebrafish [30]. The identified sHSP genes in amphibians are also orthologous to those of mammals [31]. In general, there are multiple orthologous sHSP clusters in vertebrate and angiosperm sHSPs also have similar pattern [4,32]. However, insect sHSP genes principally show one orthologous cluster that is composed of each sHSP from each species investigated, and most of the genes are species-specific (Figure 2). Thus, insect sHSPs display completely different evolutionary pattern from that of vertebrates or plants.

To determine whether the evolutionary pattern observed in five insects is universal for other arthropods and nematode, we searched the GenBank for the orthologous sHSPs in corresponding species. Indeed, we found orthologous sHSP genes in many other arthropods and nematode (Additional file 3). Most of these species are insects, such as *L. migratoria*[15], *He. erato *[33], *Ac. pisum*, and *Ae. aegypti*. These insects cover the orders Lepidoptera, Hymenoptera, Diptera, Hemiptera, Coleoptera, and Orthoptera. This ubiquitous existence in insects suggests that all of the species of insecta may possess one copy of this conserved gene in their genomes. There is a species of Arachnida, *I. scapularis*, that contains this orthologous gene. This gene is also detected within each of nine nematode species [34]. A species of chordate, *Branchiostoma lanceolatum*, also contains this orthologous sHSP gene named B1Hsp24.1 (GenBank accession number: AJ616737) with relatively lower identity [32]. Insecta and Arachnida belong to the phylum Arthropoda. Furthermore, Arthopods, Branchiostoma, and phylum Nematoda are all invertebrates. So, these orthologous sHSP genes do exist in Arthropods and Nematode and probably in all invertebrates.

That insects harbor orthologous sHSP genes is interesting. To know how these gens are related to the vertebrate sHSPs, we performed the phylogenetic analysis on the sHSPs from five insects, nematode, amphioxus, mouse and human. Although no highly supported orthologous cluster was found on the phylogenetic tree, it seems that vertebrates also have the highly conserved sHSPs that are close to insect orthologs. Therefore, it is important to understand functions of these conserved sHSPs of different organisms in future.

The orthologous sHSP genes have not only the conserved sequences but also the conserved gene structure across insect orders probably and the sequences of α-crystalling domains in these genes are more conserved than that in species-specific genes, all of which is the result of strongly purifying selection during evolution. P27, another orthologous gene of Bm21.4 in *Di. immitis*, is also constitutively expressed in male and female adult worms and developmental phase such as L3–L4 molt and is located in hypodermal tissues. Additionally, it is not up-regulated by heat shock at 43°C [34]. In *C. elegans*, Ce25 is also constitutively expressed in normal conditions and this protein is principally located in body wall muscle specially bonding to vinculin and α-actinin [35]. However, the orthologous gene in migratory locust, hsp20.6, can regulate its expression associated with the population density, for example, high density corresponding to an increased expression [15]. We infer that these orthologous sHSP genes may be constitutively expressed in organisms and may not be involved in the heat shock response. It is likely that they are house-keeping genes and important in the development, adaptability and metabolism of organisms.

Another important feature of insect sHSPs is that most of them are species-specific in five insects investigated. This suggested that functions of most sHSPs might have diverged among the insects, reflecting that the sHSPs might have contributed to adaptability of insects to different niches. Silkworm has the largest cluster of species-specific sHSPs which includes 12 genes although the cluster has a relatively low supporting value measured by posterior probability. 12 silkworm species-specific sHSPgenes diverged in sequences especially in N-terminal region (Figure 1). There are several previous studies focused on these proteins [17-19,36-38]. Silkworm sHSP genes Bm20.4, Bm20.8, Bm19.9, Bm20.1, Bm24.2, Bm23.8, and Bm19.5 have increased expression by heat shock stress [17-19,38]. It is the sHSP genes belonging to species-specific cluster that principally participate in heat shock response of silkworm and other insects. However, these genes are also expressed in usual conditions without extreme stress and have distinct expression levels in different tissues of silkworm (Figure 4). The expression of Bm19.9 is significantly high in testis and ovary [17], and Bm20.1 is highly expressed in testis of the fifth instar [38]. There are other sHSP genes having high expression intensities in gonad through the microarray data (Figure 4). Silkworm sHSP genes highly expressed in gonad may play an important role in the development of germocyte. Bm19.1, Bm15.7, and Bm23.8 are increasedly expressed in midgut. Furthermore, Bm22.6, which does not belong to the species specific cluster, is also highly expressed in midgut (Figure 4). Additionally, Bm22.6 is one of the 11 genes significantly up-regulated in midgut following the infection of BmDNV-Z, a densonucleosis virus [39]. Bm22.6 and Bm19.1 are also remarkably expressed in integument (Figure 4). Integument and midgut are tissues with immune function. Previous studies suggest that HSPs may participate in the innate immune responses of silkworm [40]. So the silkworm sHSP genes highly expressed in midgut or integument may have functions in immune defense mechanism.

There are some changes in cellular physiology before and after the heat stress. Bm20.1 principally located in nucleus and Bm20.8 in cytoplasm, are all transferred to the membrane after heat shock, and then recovered to the previous subcellular localization in normal conditions. This implies that sHSP have functions in the membrane structure and metabolism in extremely conditions [41,42]. Silkworm sHSP genes also have been studied in embryonic development of diapause and non-diapause eggs [43], silkworm parthenogenesis [44], and different silkworm breeds of bivoltine and multivoltine [45]. The studies in heat stress induced pattern, expression diversity in tissues, and other functions without chaperone of silkworm sHSP genes suggest that there are divergences in the biological functions among these genes. These species-specific sHSP genes may resist harmful stimulus for organisms and participate in the reproduction, development, and the normal metabolism activities. It is believed that the diversity and divergence of sHSP genes might have facilitated the insects adapting to various environments.

The data presented in this study revealed that the majority of insect sHSP genes in each species are located on one chromosome except for that most silkworm sHSP genes are located on two chromosomes 5 and 27. It is possible that the distribution of most silkworm sHSP genes on chromosomes 5 and 27 might be the result of split of an ancient chromosome. However, there is opposed evidence against this scenario, which is that silkworm chromosomes 5 and 28 correspond to one chromosome in *He. erato *and silkworm chromosomes 23 and 27 correspond to another one [33]. So, the expanding process of sHSP genes in silkworm is complicated. The silkworm has the most number of sHSP genes, and *An. gambiae *has the least. Whether there is relationship between the number of the sHSP genes and the resistance to thermal or other stresses is unknown. The multivoltine silkworms are more resistant than bivoltine in thermal stress, and the expressions of some sHSP genes in multivoltine breeds are lower compared with the bivoltine breeds after heat shock at 45°C [45]. The gregarious locust expresses more sHSP genes than solitary locust due to different population density, and the former have a smaller body size and a weaker fecundity [15]. Therefore, the sensitivity to heat and other stresses may influence the development and reproduction of various voltinism breeds in silkworm.

## Conclusion

Based on known silkworm sHSP sequences and the newly assembled genome sequence, we identified 16 silkworm sHSP genes. 15 of 16 silkworm sHSPs have expression evidence. The comparative analysis with other insect sHSPs revealed that there is only one orthologous cluster whereas remaining clusters are species-specific. Our data demonstrated that the evolutionary pattern of insect sHSPs which is different from that found in vertebrates and will provide some new insight into evolution of insect sHSPs. It is believed that studies on the evolution, function and mechanism of stress response genes in insects will help us understand the adaptability of insects to diverse environments.

## Methods

### Identification of insect sHSP genes

We first retrieved 6 known sHSP sequences of *B. mori *from Genbank [18] and then used these sequences as queries to search the new assembly of the silkworm genome sequence in SilkDB  for a complete list of the silkworm sHSPs. The E value for evaluating all sequences in homology search is 10-4. Finally, we obtained 16 sequences as candidate genes for the silkworm sHSPs. The other four insect annotated gene and protein sequences were downloaded from NCBI website . The four insects are *D. melanogaster*, *Ap. mellifera*, *T. castaneum*, and *An. gambiae*. We used above 16 sHSP sequences of *B. mori *as queries to perform the same homology search for other insect sHSPs as above.

One sHSP gene sequences of *S. cerevisiae *was used as the outgroup. This sequence was retrieved from Genbank (Accession no. NP_009628).

### The secondary structure and structural domains and chromosome locations of the insect sHSPs

The information of protein secondary structure and domain has advantage for helping researchers to understand the function of these proteins. So the structure information of *B. mori *candidate sHSP genes was predicted by PredictProtein , one online service tool. The prediction of protein domain was performed on InterProScan online service .

The location information of *B. mori *sHSP genes on the chromosomes was obtained by SilkMap online tool . The location information of the other four insect sHSP genes was determined by Map View tool  at NCBI website.

### Phylogenetic analysis

The amino acid sequences of sHSP genes of five insects were aligned by three methods, clustal X v.1.8, clustal W v.1.83 [46], and muscle v.3.6 [47]. The resulting alignments were almost identical and one of them was selected for further analyses. The phylogenetic tree for the insect sHSPs was first reconstructed by Neighbor-Joining (NJ) method in which distance was estimated by JTT amino acid matrix implemented in the program MEGA 4 [48]. The pairwise deletion option was used in the NJ tree reconstruction and the accuracy of the tree topology was assessed by bootstrap analysis with 1,000 resampling replicates.

Using the amino acid sequences of the conserved α-crystalling domain with 100 residues, we also reconstructed the phylogenetic relationship of the insect sHSP genes by the program MrBayes v.3.1.2 [49]. MrBayes is a program of reconstructing phylogenetic relationships based on Bayesian and maximum likelihood methods. This program simulates the posterior distribution of the phylogenetic trees through the method of Metropolis-coupled Markov Chain Monte Carlo [49]. WAG model was demonstrated to be the best model used in the MrBayes program to reconstruct the phylogenetic tree for the amino acid sequences of insect sHSPs. After MrBayes program with WAG model run 6500000 generations, the value of average standard deviation of split frequencies became stable below 0.01 and finally reached to 0.007103 at the end of the running. So, the early 3500000 generations data were seen as the burnin, which were deleted in the analysis of the results. All the trees are rooted by the sHSP gene sequence of *S. cerevisiae*, scer23.9.

### Expression information based on microarray database

*B. mori *microarray database  is composed of expression data for 22987 probes of 9 silkworm tissues on day 3 of the fifth instar in silkworm [25]. We used blastn search to obtain expression information of each silkworm sHSP gene in several tissues at normal or some stress conditions. We also searched the EST data for the silkworm sHSP genes in SilkDB to confirm whether these genes identified from the genome search approach are expressed in the organism or not .

## Authors' contributions

ZZ made the study design. ZWL did the data collection and analysis, and drafted the manuscript. XL and QYY read the manuscript. HK and ZZ revised the manuscript. ZZ and ZHX supervised the study. All authors read and approved the final manuscript.

## Supplementary Material

Additional file 1 **Graphics of insect tandem sHSP genes**. Graphics for tandem arrangements of sHSP genes in respective insect genomes. Click here for file

Additional file 2 **The alignment of amino acid sequences used to reconstruct the phylogenetic trees in Figure **2. The alignment of amino acid sequences used to reconstruct the phylogenetic trees in Figure 2. Click here for file

Additional file 3 **Phylogenetic tree of invertebrate sHSPs**. The phylogenetic tree is constructed with the whole amino acid sequences of five insect sHSP genes and the orthologous sHSP genes. Program MAGE 4.0 with NJ method is used to construct this tree. The orthologous sHSP genes are contained in the "orthologous cluster" Click here for file

Additional file 4 **The phylogenetic tree based on the sHSP genes of insects, nematode, amphioxus, mouse and human**. We reconstructed the tree using MEGA 4 with JTT amino acid matrix and 1000 bootstrap replicates. The accession numbers of sHSP genes in nematode (e.g., Ce12.1), mouse (e.g., MmusHSPB1–11) and human (e.g., HsapHSPB1–11) are acquired from previous studies [5,42,43]. In addition, we searched the genome of an amphioxus, *Branchiostoma floridae*, using blast method, and obtained 14 putative sHSP genes. They are Bflo130908(XM_002242495), Bflo282111(XM_002231495), Bflo127262(XM_002231460), Bflo130907XM_002242478), Bflo252489(XM_002246138), Bflo151636(XM_002246139), Bflo237499(XM_002237229), Bflo151683(XM_002246121), Bflo126126(XM_002228486), Bflo91065(XM_002228487), Bflo122123(XM_002216227), Bflo91211(XM_002228634), Bflo198433(XM_002203251), Bflo202574(XM_002203246). Click here for file
