# Supplementary figures and images for: The small heat shock protein (sHSP) genes in the silkworm, Bombyx mori, and comparative analysis with other insect sHSP genes
Source: BMC Evol Biol. 2009 Aug 28;9:215. doi: 10.1186/1471-2148-9-215 (PMC2745388; doi:10.1186/1471-2148-9-215)

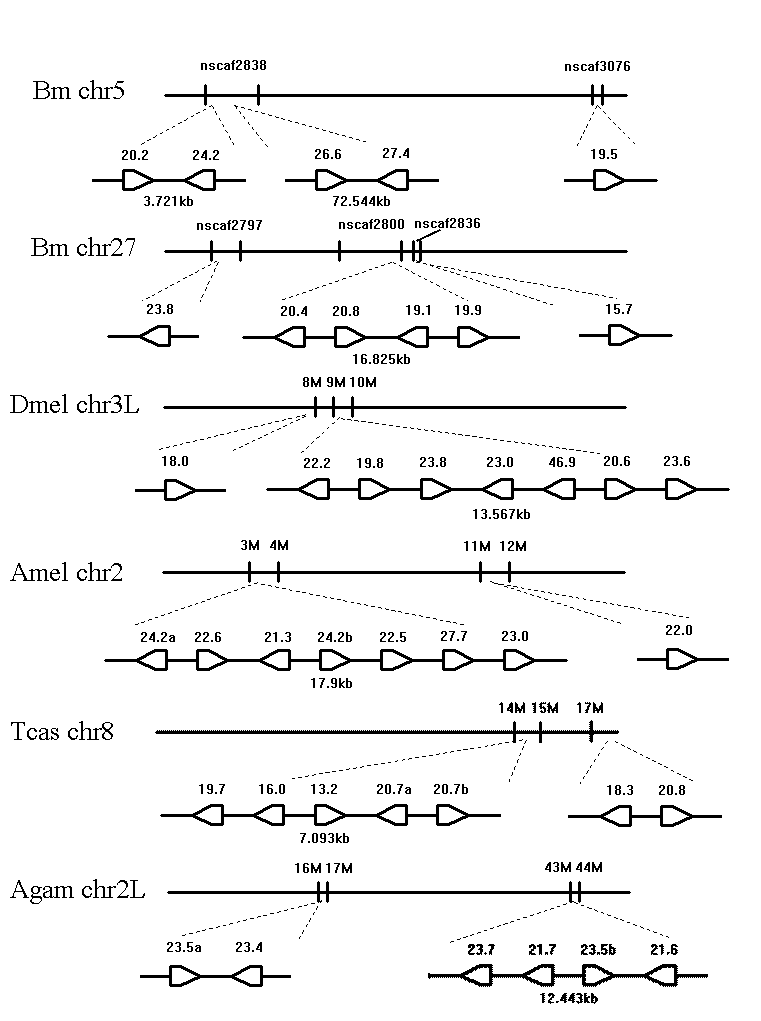


.

Supplement: Additional file 1 — Graphics of insect tandem sHSP genes. Graphics for tandem arrangements of sHSP genes in respective insect genomes. [file 1471-2148-9-215-S1.doc]

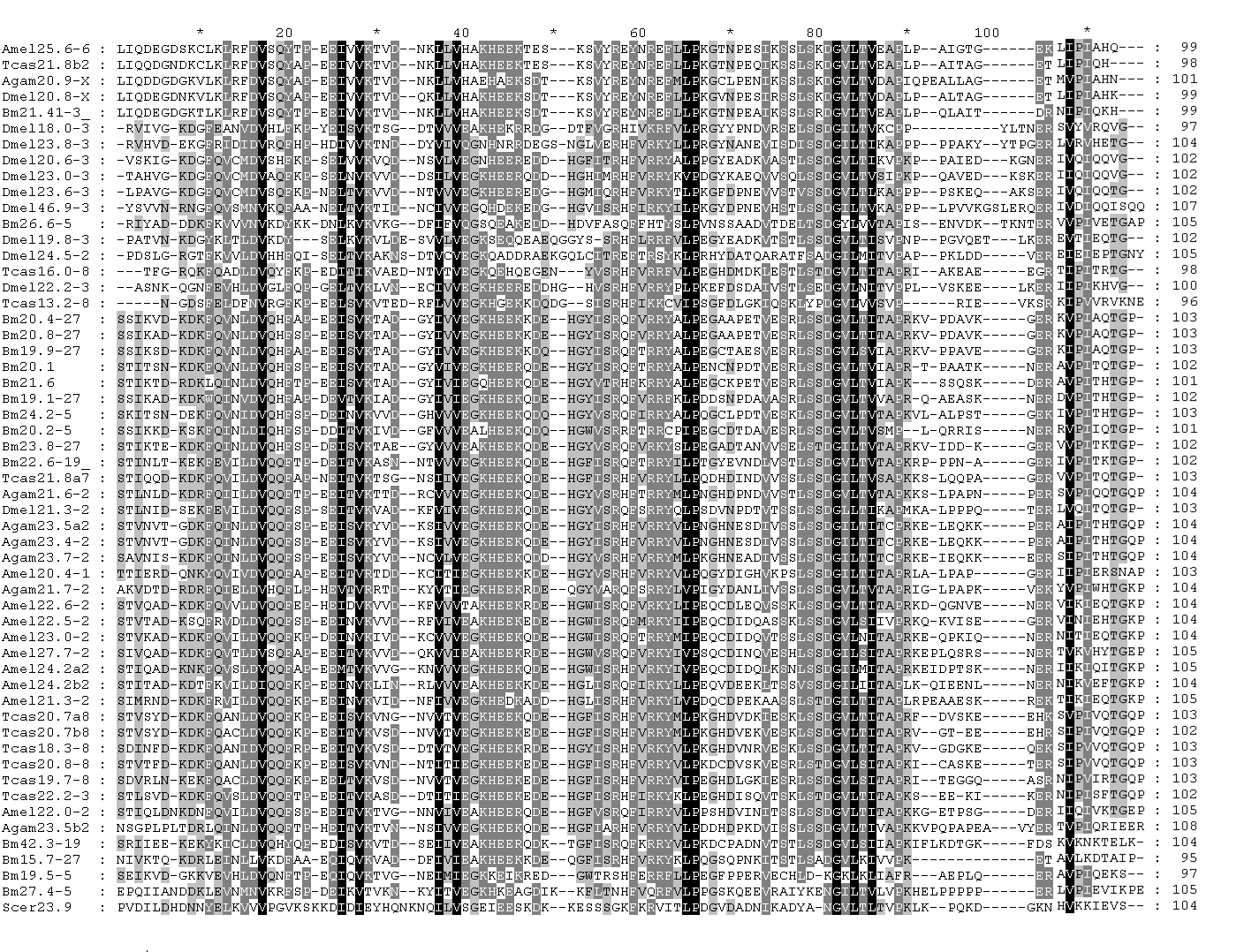

Supplement: Additional file 2 — The alignment of amino acid sequences used to reconstruct the phylogenetic trees in Figure 2. The alignment of amino acid sequences used to reconstruct the phylogenetic trees in Figure 2. [file 1471-2148-9-215-S2.doc]

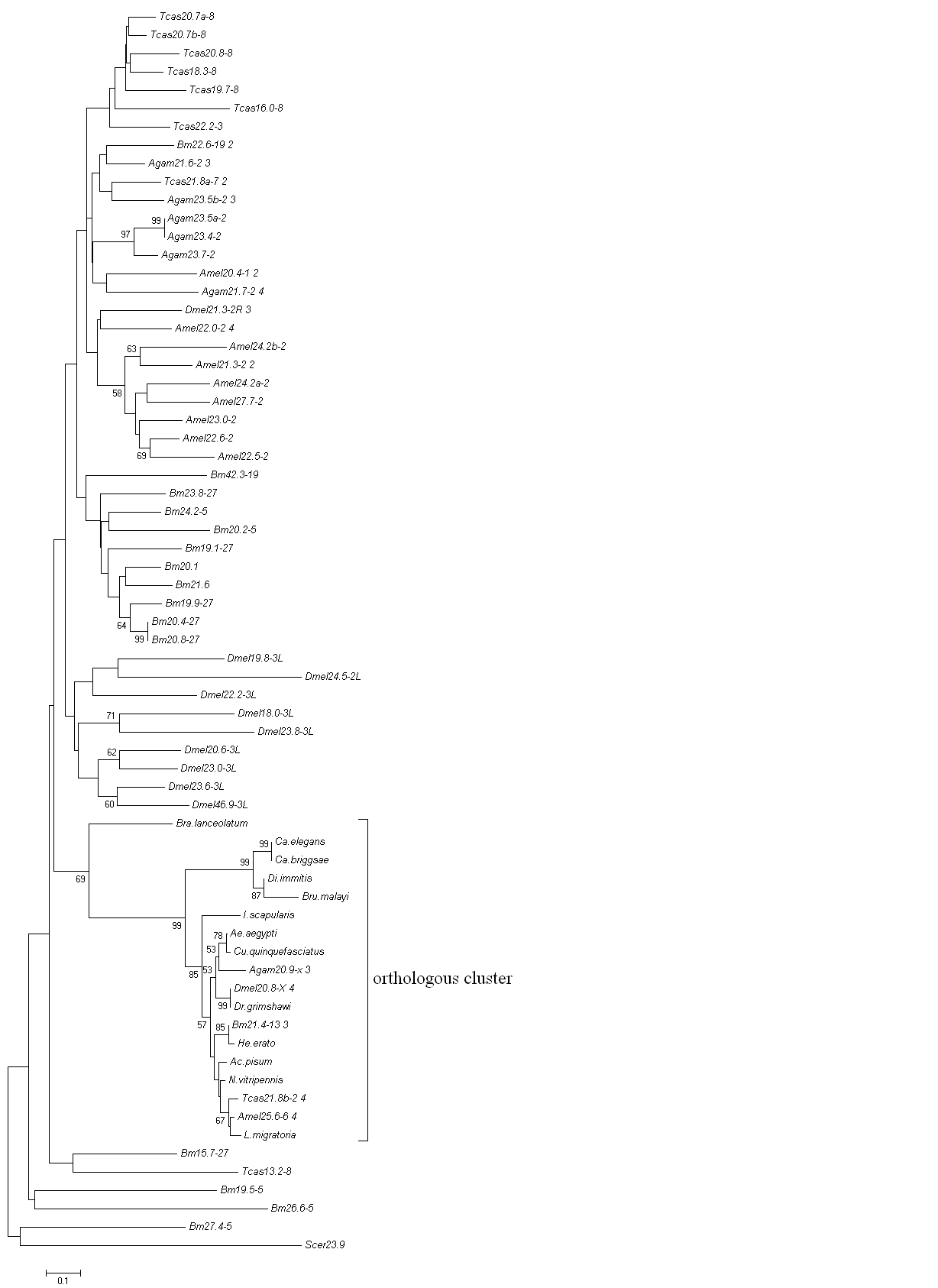

Supplement: Additional file 3 — Phylogenetic tree of invertebrate sHSPs. The phylogenetic tree is constructed with the whole amino acid sequences of five insect sHSP genes and the orthologous sHSP genes. Program MAGE 4.0 with NJ method is used to construct this tree. The orthologous sHSP genes are contained in the "orthologous cluster" [file 1471-2148-9-215-S3.doc]
