# Supplementary material for: The small heat shock protein (sHSP) genes in the silkworm, Bombyx mori, and comparative analysis with other insect sHSP genes
Source: BMC Evol Biol. 2009 Aug 28;9:215. doi: 10.1186/1471-2148-9-215 (PMC2745388; doi:10.1186/1471-2148-9-215)
Supplement: Supplementary file 2 [file 1471-2148-9-215-S4.doc]

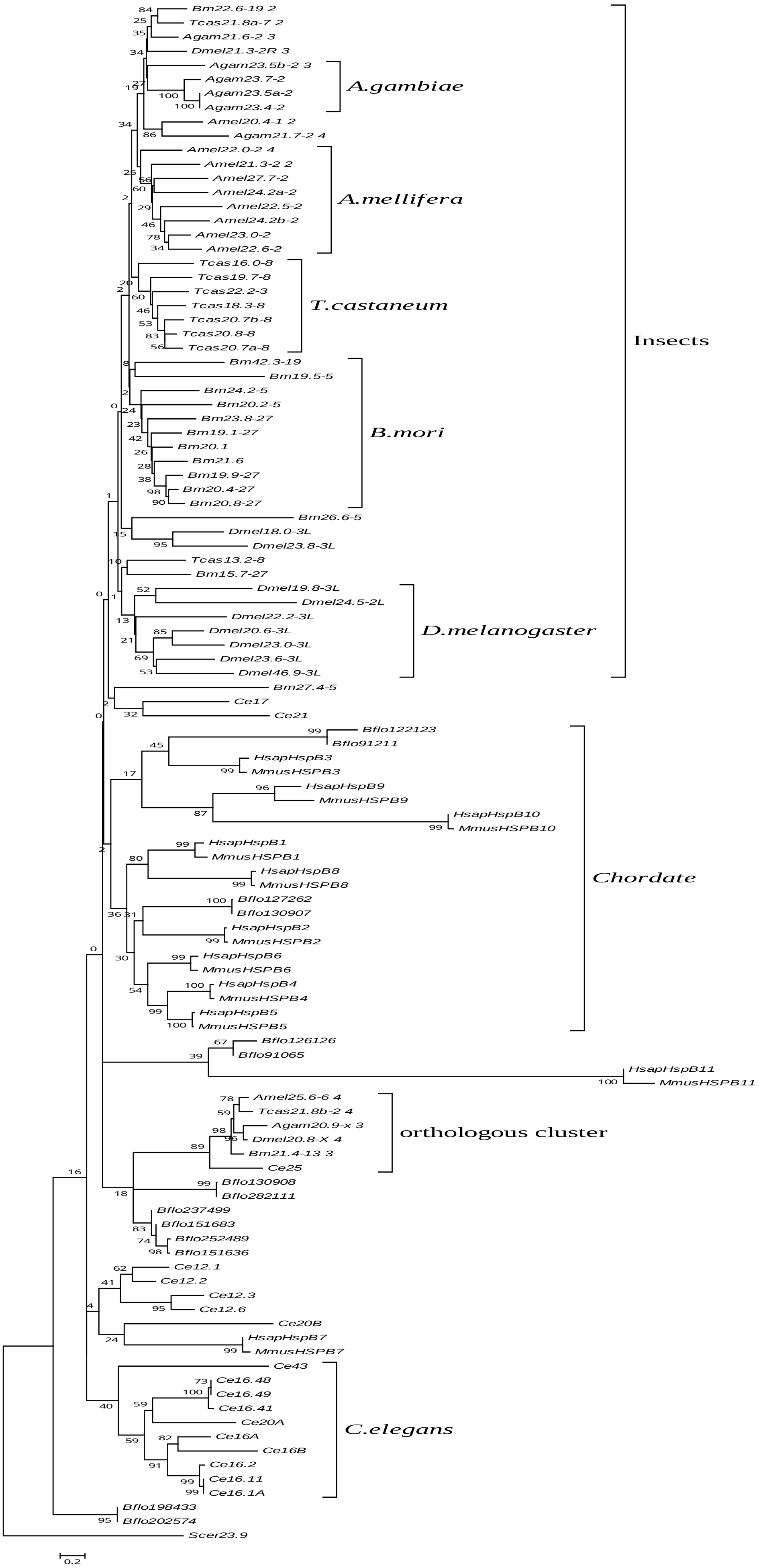

Bm22.6-19 2
Tcas21.8a-7 2
Agam21.6-2 3
Dmel21.3-2R 3
Agam23.5b-2 3
Agam23.7-2
Agam23.5a-2
Agam23.4-2
A.gambiae
Amel20.4-1 2
Agam21.7-2 4
Amel22.0-2 4
Amel21.3-2 2
Amel27.7-2
Amel24.2a-2
Amel22.5-2
Amel24.2b-2
Amel23.0-2
Amel22.6-2
A.mellifera
Tcas16.0-8
Tcas19.7-8
Tcas22.2-3
Tcas18.3-8
Tcas20.7b-8
Tcas20.8-8
Tcas20.7a-8
T.castaneum
Bm42.3-19
Bm19.5-5
Bm24.2-5
Bm20.2-5
Bm23.8-27
Bm19.1-27
Bm20.1
Bm21.6
Bm19.9-27
Bm20.4-27
Bm20.8-27
B.mori
Bm26.6-5
Dmel18.0-3L
Dmel23.8-3L
Tcas13.2-8
Bm15.7-27
Dmel19.8-3L
Dmel24.5-2L
Dmel22.2-3L
Dmel20.6-3L
Dmel23.0-3L
Dmel23.6-3L
Dmel46.9-3L
D.melanogaster
Insects
Bm27.4-5
Ce17
Ce21
Bflo122123
Bflo91211
HsapHspB3
MmusHSPB3
HsapHspB9
MmusHSPB9
HsapHspB10
MmusHSPB10
HsapHspB1
MmusHSPB1
HsapHspB8
MmusHSPB8
Bflo127262
Bflo130907
HsapHspB2
MmusHSPB2
HsapHspB6
MmusHSPB6
HsapHspB4
MmusHSPB4
HsapHspB5
MmusHSPB5
Chordate
Bflo126126
Bflo91065
HsapHspB11
MmusHSPB11
Amel25.6-6 4
Tcas21.8b-2 4
Agam20.9-x 3
Dmel20.8-X 4
Bm21.4-13 3
Ce25
orthologous cluster
Bflo130908
Bflo282111
Bflo237499
Bflo151683
Bflo252489
Bflo151636
Ce12.1
Ce12.2
Ce12.3
Ce12.6
Ce20B
HsapHspB7
MmusHSPB7
Ce43
Ce16.48
Ce16.49
Ce16.41
Ce20A
Ce16A
Ce16B
Ce16.2
Ce16.11
Ce16.1A
C.elegans
Bflo198433
Bflo202574
Scer23.9
0.2
